# A Fast and Quantitative Method for Post-translational Modification and Variant Enabled Mapping of Peptides to Genomes

**DOI:** 10.3791/57633

**Published:** 2018-05-22

**Authors:** Christoph N. Schlaffner, Georg J. Pirklbauer, Andreas Bender, Judith A.J. Steen, Jyoti S. Choudhary

**Affiliations:** ^1^Department of Neurobiology, F. M. Kirby Neurobiology Center, Boston Children's Hospital, Harvard Medical School; ^2^Proteomic Mass Spectrometry, Wellcome Trust Sanger Institute, Wellcome Genome Campus; ^3^Centre for Molecular Informatics, Department of Chemistry, University of Cambridge; ^4^Functional Proteomics Group, Chester Beatty Laboratories, Institute of Cancer Research

**Keywords:** Genetics, Issue 135, proteomics, genomics, open-source software, annotation, proteogenomics, mapping, visualization, genome browser, large-scale, track-hubs

## Abstract

Cross-talk between genes, transcripts, and proteins is the key to cellular responses; hence, analysis of molecular levels as distinct entities is slowly being extended to integrative studies to enhance the understanding of molecular dynamics within cells. Current tools for the visualization and integration of proteomics with other omics datasets are inadequate for large-scale studies. Furthermore, they only capture basic sequence identify, discarding post-translational modifications and quantitation. To address these issues, we developed PoGo to map peptides with associated post-translational modifications and quantification to reference genome annotation. In addition, the tool was developed to enable the mapping of peptides identified from customized sequence databases incorporating single amino acid variants. While PoGo is a command line tool, the graphical interface PoGoGUI enables non-bioinformatics researchers to easily map peptides to 25 species supported by Ensembl genome annotation. The generated output borrows file formats from the genomics field and, therefore, visualization is supported in most genome browsers. For large-scale studies, PoGo is supported by TrackHubGenerator to create web-accessible repositories of data mapped to genomes that also enable an easy sharing of proteogenomics data. With little effort, this tool can map millions of peptides to reference genomes within only a few minutes, outperforming other available sequence-identity based tools. This protocol demonstrates the best approaches for proteogenomics mapping through PoGo with publicly available datasets of quantitative and phosphoproteomics, as well as large-scale studies.

**Figure:**
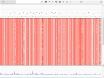


## Introduction

In cells, genome, transcriptome, and proteome affect each other to modulate a response to internal and external stimuli and interact with each other to carry out specific functions leading to health and disease. Therefore, characterizing and quantifying genes, transcripts, and proteins is crucial for fully understanding cellular processes. Next-generation sequencing (NGS) is one of the most commonly applied strategies to identify and quantify gene and transcript expression. However, protein expression is commonly assessed by mass spectrometry (MS). Significant advancements in MS technology over the last decade has enabled more a complete identification and quantification of proteomes, making the data comparable with transcriptomics^1^. Proteogenomics and multi-omics as ways to integrate NGS and MS data have become powerful approaches to assess cellular processes across multiple molecular levels, identifying subtypes of cancer and leading to novel potential drug targets in cancer^2^,^3^. It is important to note that proteogenomics was initially used to provide proteomic evidence for gene and transcript annotations^4^. Several genes previously thought to be non-coding have recently undergone reevaluation considering large-scale human tissue datasets^5^,^6^,^7^. In addition, proteomic data are successfully used to support annotation efforts in non-model organisms^8^,^9^. However, proteogenomic data integration can be exploited further to highlight protein expression in relation to genomic features and elucidate cross-talk between transcripts and proteins by providing a combined reference system and methods for co-visualization.

In order to provide a common reference for proteomics, transcriptomics, and genomics data, numerous tools have been implemented for mapping peptides identified through MS onto genome coordinates^10^,^11^,^12^,^13^,^14^,^15^,^16^,^17^. Approaches differ in aspects such as mapping reference, support of genome browsers, and degree of integration with other proteomics tools as shown in Figure 1. While some tools map reverse translated peptides onto a genome^16^, others use a search engine annotated position within a protein and gene annotation to reconstruct the nucleotide sequence of the peptide^15^. Still others use a 3- or 6-frame translation of the genome to map peptides against^11^,^13^. Lastly, several tools skip the nucleotide sequences and use amino acid sequence translations from RNA-sequencing mapped transcripts as an intermediate to map peptides to the associated genome coordinates^10^,^12^,^14^,^17^. However, the translation of nucleotide sequences is a slow process and custom databases are prone to errors that propagate to the peptide mapping. For fast and high-throughput mapping, a small and comprehensive reference is crucial. Therefore, a standardized protein reference with associated genome coordinates is essential for accurate peptide to genome mapping. Novel aspects in proteogenomics, such as the incorporation of variants and post-translational modifications (PTMs)^2^,^3^, are gaining momentum through recent studies. However, these are generally not supported by current proteogenomic mapping tools as shown in Figure 1. To improve speed and quality of mapping, PoGo was developed, a tool that allows the fast and quantitative mapping of peptides to genomes^18^. In addition, PoGo enables the mapping of peptides with up to two sequence variants and annotated post-translational modifications.

PoGo has been developed to cope with the rapid increase of quantitative high-resolution datasets capturing proteomes and global modifications and provides a central utility for large-scale analyses such as personal variation and precision medicine. This article describes the application of this tool to visualize the presence of post-translational modification in relation to genomic features. Furthermore, this article highlights the identification of alternative splicing events through mapped peptides and the mapping of peptides identified through custom variant databases to a reference genome. This protocol employs publicly available datasets downloaded from the PRIDE archive^19^ to demonstrate these functionalities of PoGo. In addition, this protocol describes the application of TrackHubGenerator for the creation of online accessible hubs of peptides mapped to genomes for large-scale proteogenomics studies.

## Protocol

### 1. Preparation, Download, and Setup

NOTE: The file and folder path examples are shown in a Windows format for the ease of access for standard users. PoGo and PoGoGUI are also available for macOS and Linux operating systems.

1. **Download PoGo and PoGoGUI from GitHub**
  1. Open a web browser and navigate to PoGo on GitHub (http://github.com/cschlaffner/PoGo/). Select **Releases** and download the latest release zip compressed file. Extract the compressed file into the executables folder (*e.g.*, C:\PoGo\executables\).
  2. Navigate in the web browser to PoGoGUI on GitHub (http://github.com/cschlaffner/PoGoGUI/). Select **Releases **and download the latest release jar file (*e.g.,* “PoGoGUI-v1.0.2.jar”). Store the jar file in the executables folder.
2. **Download the genome annotation and translated protein-coding sequences** NOTE: Download the genome annotation and translated protein-coding sequences for supported species from GENCODE^7^ (www.gencodegenes.org) or Ensembl^20^ (www.ensembl.org) in the General Transfer Format (GTF) and the protein sequences in FASTA format.
  1. In the web browser, navigate to www.gencodegenes.org and select **Data | Human | Current Release**. Download the **Comprehensive gene annotation** via the GTF link and extract the gz-compressed file into the data folder (*e.g.*, C:\PoGo\Data\) using an unzipping program (*e.g.*, 7-Zip).
  2. Download the **Protein-coding transcript translation sequences** via the FASTA link and extract the gz-compressed file into the data folder generated in the previous step.
    1. Alternatively, navigate in the web browser to www.ensembl.org and select** Downloads** followed by **Download data via FTP**. Find a supported species (*e.g.*, Human). Download the latest release file for transcript annotation using the GTF link in the **Gene set** column. Choose the file with name structure “species.release.gtf.gz” and extract the gz-compressed file into the data folder.
  3. Download the latest release protein-coding transcript translation sequences using the FASTA link in the **Protein sequence (FASTA)** column. Choose the file with the name structure “species.release.pep.all.fa.gz” and extract the gz-compressed file into the data folder.
3. **Prepare peptide identification files ** NOTE: PoGo only supports a 4-column format containing sample identifier, peptide sequence, number of peptide-spectrum-matches (PSMs), and quantitative value. However, PoGoGUI supports standardized identification file formats mzIdentML, mzid, and mzTab, and converts them into PoGo’s 4-column format using the publicly available framework ms-data-core-api^21^. Files in mzIdentML, mzid, or mzTab format can be downloaded from the PRIDE archive^19^. Alternatively, data can be provided in a tab-separated file format with the extension .tsv or .pogo. The format contains 4 columns with the following column headers: sample identifier (Sample), peptide sequences (Peptide), number of peptide-spectrum-matches (PSMs), and peptide quantitation (Quant). An example is shown in **Figure 2**.
  1. Download an example file in mzTab format from a proteomics study on human testis from the PRIDE archive^19^ (https://www.ebi.ac.uk/pride/archive/projects/PXD006465/files^22^).
  2. Save and extract the gz-compressed file into the data folder created in step 1.2.1. NOTE: Alternatively, download example data for human phosphoproteomics searched with MaxQuant from the PRIDE archive (file “Traktman_2013_MaxQuantOutput-full.zip” from https://www.ebi.ac.uk/pride/archive/projects/PXD005246/files^23^).
  3. Save and extract the zip-compressed file in the data folder that was created in step 1.2.1.
  4. Open a blank spreadsheet and import the peptides.txt file from the folder C:/PoGo/Data/Traktman_2013_MaxQuantOutput-full/combined/txt/ using the option **Data | From Text/CSV**. In the opening window, click **Edit**.
  5. Remove all columns with the exception of “Sequence”, “Experiment BR1”, “Experiment BR2”, “Experiment BR3”, “Ratio H/L normalized BR1”, “Ratio H/L normalized BR2”, and “Ratio H/L normalized BR3”.
  6. Select the columns “Ratio H/L normalized BR1”, “Ratio H/L normalized BR2”, and “Ratio H/L normalized BR3” and click **Transform | Unpivot Columns**. Select the columns “Experiment BR1”, “Experiment BR2”, and “Experiment BR3” and repeat the unpivot operation.
  7. Select the resulting column “Attribute” and split the contents using **Transform | Split Column | By Delimiter**. Select **Space** as delimiter in the drop-down menu. Repeat the operation for column “Attribute.1”.
  8. Remove the resulting columns “Attribute.1.1”, “Attribute.2”, “Attribute.3”, and “Attribute.1.1.1”.
  9. Add a column by using the **Add Column | Custom Column** option. Adapt the custom column formula to represent the following: “=[Attribute.4]=[Attribute.1.2]”.
  10. Apply a filter to the generated custom column to filter out all lines containing “FALSE”; only lines containing “TRUE” will remain.
  11. Remove the columns “Attribute.1.2” and “Custom” and change the order of the remaining columns to the following: “Attribute.4”, “Sequence”, “Value.1”, and “Value”.
  12. Change the column names to “Experiment”, “Peptide”, “PSMs”, and “Quant”, respectively. Load the file using **Home | Close&Load**.
  13. Save the file as a tab-delimited file using **File | Save As** and select the type “Text (Tab delimited) (*.txt)”. Change the name to “peptides_pogo.txt” and save it in the folder C:/PoGo/Data.

### 2. Mapping Peptides with Annotated Post-translational Modifications and Visualization Including Quantitation

NOTE: The resulting output file can be loaded in any genome browser supporting Browser Extensible Data (BED) format. A selection of browsers is the Integrative Genome Browser (IGV)^24^ (which is used in the following), the UCSC Genome Browser^25^, and the Ensembl Genome Browser^20^. It is important to note that the annotation GTF and protein FASTA versions used for PoGo mapping match the version of the genome in the genome browser. For the human Ensembl releases 57-75 and GENCODE versions 3d-19, use GRCh37/hg19; for the Ensembl versions 76 or higher and GENCODE 20 or higher, use GRCh38/hg38. For the mouse Ensembl versions 74 or higher and GENCODE M2 or higher, use GRCm38.

1. **Map peptides using PoGoGUI (see Figure 3).**
  1. Navigate to the executables folder. Start the program by double-clicking the icon **PoGoGUI-vX.X.X.jar**. NOTE: The graphical user interface will start up and allow easy and visual selection of options.
  2. Use the **Select** button next to the “PoGo Executable”. Then, navigate in the executables folder to the relevant operating systems subfolder (*e.g.*, C:\PoGo\Executables\Windows\). Select the executable of PoGo (*e.g.*, PoGo.exe) and confirm its selection by clicking the **Open** button.
  3. Select the reference input file for protein sequences by clicking **Select**. Navigate to the data folder and select the translation FASTA file. Confirm its selection by clicking the **Open** button.
  4. Select the transcript annotation file using the **Select** button. Navigate to the data folder and select the annotation GTF file. Confirm the selection by clicking the **Open** button.
  5. Add the peptide identification file—multiple file selection is enabled—by using the **Add** button next to “Peptide Files”. Select a file in the supported format mzTab, mzIdentML, or mzid, or in the tab-separated 4-column format downloaded and prepared in step 1.3.
  6. Untick the checkboxes next to BED and GTF in the output formats selection. Only leave PTM BED and GCT checked.
  7. Select the appropriate species for the data from the drop-down selection. It is essential that the FASTA file, the GTF file, and the drop-down selection are for the same species.
  8. Start mapping by clicking the **START** button. NOTE: If necessary, PoGoGUI will convert the input file into pogo format, provide the pogo files in the same folder for future convenience, and start the mapping process. The conversion of a single mzTab file downloaded in step 1.3.1 will last between 10 - 20 min before mapping commences.
2. **Visualizing in the integrative genomics viewer** NOTE: See** Figure 4**.
  1. Load the PoGo output file ending in “_ptm.bed” in the IGV through **File | Load from File** and select the file. NOTE: Due to size, some files may require the generation of an index to allow a quick reloading of the genomic regions. The IGV will prompt the user automatically to the generation. Follow the instructions indicated.
  2. Repeat the loading step for the file ending in “_noptm.bed”. This file contains all peptides found without any modification.
  3. Note that each loaded file will be shown as separate tracks with the file name identifying the track. Reorder tracks by dragging and dropping them to the desired position in the list.
  4. Note that each track is initially shown in a collapsed manner. To expand them, right click on the track name and select either expanded for a full view of the peptides including the sequences or squished for a stacked view.
  5. Repeat the loading step for the file ending in “.gct”. This file contains the peptide quantitation per annotated sample.
  6. Unlike for the files loaded above, each annotated sample will be loaded as a separate track. Reorganize the samples through drag and drop operations.
  7. Navigate within the genome by selecting a chromosome in the drop-down menu, type in genomic coordinates, search a gene symbol, or click and hold to select a section of a chromosome to zoom in.

### 3. Mapping Peptides Identified Through a Custom Variant Database to a Reference Genome

NOTE: PoGo mapping can be carried out using the graphical user interface (GUI) or through the command line interface. They are interchangeable. In this part of the protocol, the command line interface is used to highlight interchangeability. The second part of this protocol section requires the software tool R^26^. Please ensure that the package is installed.

1. Map the reference peptides to the reference genome.
  1. Open a command prompt (cmd) and navigate to the executables folder of PoGo (*e.g.*, C:\PoGo\Executables\).
  2. Type the command below: PoGo.exe -gtf \PATH\TO\GTF -fasta \PATH\TO\FASTA -in \PATH\TO\IN -format BED -species MYSPECIES
    1. Substitute the \PATH\TO\GTF, \PATH\TO\FASTA, and \PATH\TO\IN with paths to the annotation GTF, protein sequence FASTA, and peptide identification file (in the 4-column format with file ending “.tsv” or “.pogo”) respectively. Also substitute MYSPECIES with the species consistent with the data (*e.g.,* Human).
  3. Confirm the execution by pressing the “Enter” key. Wait till the execution is finished before progressing any further. NOTE: This may take a few minutes. The resulting file will be stored in the same folder as the peptide input file and will be regarded as \PATH\TO\OUT.pogo.bed in the following.
2. Extract only variant peptides from the input file.
  1. Open R and load the input file \PATH\TO\IN using the following command: inputdata <- read.table(“PATH/TO/IN”,header=TRUE,sep=”\t”)
  2. Load the already mapped peptides using the command: mappedpeptides <- read.table(“PATH/TO/OUT.pogo.bed”,sep=”\t”,header=FALSE)
  3. Remove peptides that were already mapped from the inputdata: peptidesnotmapped <- inputdata[!(inputdata$Peptide %in% mappedpeptides$V4),]
  4. Print the unmapped peptides into a new input file: write.table(peptidesnotmapped, “PATH\TO\IN.notmapped.pogo”, header=FALSE, sep=”\t”, col.names=TRUE,row.names=FALSE,quote=FALSE)
3. Map the remaining peptides to the reference genome allowing mismatches.
  1. As in step 3.1, open the command prompt and navigate to the executables folder of PoGo.
  2. Type the command below allowing 1 amino acid mismatch and substitute the \PATH\TO\GTF, \PATH\TO\FASTA, and \PATH\TO\IN.notmapped.pogo with paths to the annotation GTF, protein sequence FASTA, and peptide identification file created in step 3.2. Also substitute MYSPECIES with the species consistent with the data (*e.g.*, Human).
    1. PoGo.exe -gtf \PATH\TO\GTF -fasta \PATH\TO\FASTA -in \PATH\TO\IN -format BED -species MYSPECIES -mm 1
  3. Confirm the command’s execution by pressing the “Enter” key. Wait till the execution is finished before progressing any further. NOTE: This may take a few minutes. The resulting file will be stored in the same folder as the peptide input file and will be regarded as \PATH\TO\OUT.pogo_1MM.bed in the following.
4. Visualize the peptides mapped without and with mismatch in the IGV as described in step 2.2.

### 4. Mapping Using Multiple Files and Generating Track Hubs for Large Datasets

1. **Mapping peptides from multiple files using PoGoGUI**
  1. Navigate to the executables folder and start the program GUI by running **PoGoGUI-vX.X.X.jar**.
  2. Select the PoGo executable file for the operating system in use (here Linux), as well as the reference input protein sequences FASTA file and the annotation GTF file as described in protocol steps 2.1.2 - 2.1.4.
  3. Add the peptide identification files by using the **Add** button next to “Peptide Files”; multiple file selection is enabled, as well as drag-and-drop into the blank field underneath “Peptide Files”.
  4. Untick the checkboxes next to PTM BED, GTF, and GCT in the output formats section and only leave BED checked.
  5. Select the option **Merge multiple input files into single output**. NOTE: This will result in a single output file combining all peptides of the input files. Leaving this option unselected will result in a sequential execution of the program for each input file separately.
  6. Select the appropriate species for the data from the drop-down selection consistent with the FASTA and GTF files.
  7. Start mapping by clicking the **START** button. If necessary, the program will convert the input files into pogo format. This might take some time to execute. In the meantime, download the required tools and scripts for the track hub generation.
2. **Preparing for track hub generation**
  1. Open a web browser, navigate to https://github.com/cschlaffner/TrackHubGenerator and download the file “TrackHubGenerator.pl”. Save the file to the executables folder.
  2. In the web browser, navigate to www.hgdownload.soe.ucsc.edu/admin/exe/ and select the folder for the operating system in use (here Linux). Download the tool **bedToBigBed** and the script **fetchChromSizes** into the executables folder^27^.
3. **Generating a track hub from mapped peptides** NOTE: After PoGoGUI has finished mapping the peptides, a track hub can be automatically generated for all the resulting files in BED format stored in the same folder.
  1. Open a terminal window and type the following command: Perl TrackHubGenerator.pl PATH/TO/NAME ASSEMBLY FBED UCSC EMAIL
    1. Substitute PATH/TO/NAME with a file path and name for the track hub (*e.g.*, ~/PoGo/Data/Mytrackhub), ASSEMBLY with the genome assembly on which the annotation is based (*e.g.*, hg38 for human), FBED with the path to the folder containing the BED files on which the track hub will be based (*e.g.*, ~/PoGo/Data/), UCSC with the folder where the tools downloaded from UCSC are stored (*e.g.*, ~/PoGo/Executables/), and EMAIL with an email address for the person responsible for the track hub.
  2. Confirm the execution by pressing the “Enter” key; the execution will only take a short time to finish.
  3. Transfer the generated track hub (*i.e.*, the created folder ~/PoGo/Data/Mytrackhub/) with all its contents to a web-accessible FTP server. NOTE: An FTP server with an associated web server enabling access to the track hub via the protocols ftp and http is preferred. The repositories github (github.com) and figshare (figshare.com) support this type of access and can be used instead of a FTP server.
4. **Visualizing a track hub in the UCSC genome browser**
  1. In a web browser, navigate to https://genome.ucsc.edu/ and select **MyData | Track Hubs**. Click on the tab **My Hubs**.
  2. Copy the URL to the track hub into the text field. NOTE: The URL consists of the server address, the track hub location and name, and the hub.txt file (*e.g.*, http://ngs.sanger.ac.uk/production/proteogenomics/WTSI_proteomics_PandeyKusterCutler_tissues_hi/hub.txt).
  3. Load the track hub by clicking **Add Hub**. NOTE: The hub will be loaded, and a short message will appear, stating details of the track hub such as its name, the contact information of the person responsible for the track hub, and the genome assembly used. The website will return to the main page.
  4. Select **GenomeBrowser** to enter the browser view. NOTE: The custom track hub will be shown at the top of the list. If multiple BED files built the basis for the track hub, each of the files will be represented as a separate track within the hub.

## Representative Results

A graphical depiction highlighting at which stage of a regular proteomic workflow PoGo^18^ is applied, as well as downstream options of visualization, is shown in Figure 5. Shotgun proteomics (*i.e.*, the proteolytic digestion of proteins followed by liquid chromatography coupled with tandem mass spectrometry) is one precursory step of proteogenomic mapping. The resulting tandem mass spectra are commonly compared to theoretical spectra derived from protein sequence databases. Proteogenomics studies introduce translation sequences of novel transcripts with coding potential and non-synonymous single nucleotide variants (SNVs) into the database, making it hard to easily relate these back to the reference genome^8^. The graphical user interface of PoGo (PoGoGUI) supports file formats for the standardized reporting of peptide identifications from mass spectrometry experiments and converts them into the simplified 4-column pogo format. PoGoGUI wraps the command line tool PoGo and thus enables the mapping of peptides onto genome coordinates utilizing the reference annotation of protein-coding genes commonly provided in the GTF and the translated transcript sequences in FASTA format. Different output formats are generated by PoGo to enable the visualization of different aspects of the peptides identified through mass spectrometry, including post-translational modifications and peptide level quantification. Output files in the BED can further be converted and combined into online accessible directories called track hubs. Single output files, as well as track hubs, then can be visualized in browsers such as the UCSC Genome Browser^25^, Ensembl Genome Browser^20^, IGV^24^, and Biodalliance^28^ (see Figure 5 bottom).

We applied PoGo to the reanalysis of the draft human proteome maps filtered at high significance as described in Wright *et al.*^7^ and compared it to two other tools for proteogenomic mapping, namely iPiG^14^ and PGx^10^. The dataset comprised 233,055 unique peptides across 59 adult and fetal tissues resulting in a total of over 3 million sequences. PoGo outperformed these tools both in runtime (6.9x and 96.4x faster, respectively) and memory usage (20% and 60% less memory, respectively) as shown in Figure 6^18^. An example of a successfully mapped peptide is shown in Figure 7.

While PoGo significantly outperformed the other tools in speed and memory, it also is capable of mapping post-translational modifications and quantitative information associated with peptides onto the genome. Figure 8A schematically depicts the visualization of the BED format in a genome browser for peptides mapping to one exon and across splice junctions. PoGo utilizes the coloring option to provide easy visual aid with respect to the uniqueness of the peptide mapping within the genome. Mappings in red indicate uniqueness to a single transcript, while black highlights mapping to a single gene. However, the peptide is shared between different transcripts. Grey mappings show a peptide shared between multiple genes. These are, for example, less reliable for the quantification of a gene or untrustworthy to call the expression of a gene. The PTM BED option of PoGo redefines the color code to accommodate different types of post-translational modifications as shown in Figure 8B. Additionally, PTMs are indicated by thick blocks (see Figure 8B). A single PTM of a type is highlighted by a thick block at the position of the modified amino acid residue, while multiple PTMs of the same type are spanned by a thick block from the first modified amino acid to the last.

We applied PoGo and subsequently TrackHubGenerator to a dataset of 50 colorectal cancer cell lines including whole proteome and phosphoproteome^29^. While the track hub loaded in the UCSC Genome Browser shows the peptides mapped to the genome and highlights the uniqueness of the mappings and the phosphorylation sites (see Figure 9), additional data are provided in the supplemental folder. The GCT files then enable the visualization of the peptide and phosphopeptide quantitation in a genomic context. However, GCT files do not provide an easy visualization of peptides spanning across splice junctions (see Figure 10 top). The peptides across splice junctions are split into their respective parts mapping to the exons. While it is possible to identify splice peptides through the same quantitative values of exon mappings, loading sequence-based mapping files such as BED or GTF that connect the exons by a thin intron spanning line support the interpretation (see Figure 10 bottom).

To highlight the utility of variant enabled mapping, we applied PoGo in two configurations to a dataset of human testis proteome searched against neXtProt to hunt for missing proteins using a multi-enzyme strategy^22^. The neXtProt comprises besides reference protein sequences over 5 million single amino acid variants^30^. Mapping peptides identified with a single amino acid variant is not supported by other mapping tools. A total of 177,012 unique peptides were identified. Of these, 99.8% (176,694) peptides were first successfully mapped without allowing mismatches. Removing those from the identified peptide list resulted in 0.2% (318) peptides that subsequently were mapped allowing one amino acid substitution. This resulted in 3,446 mappings of 162 peptides that would not have been mapped to the reference genome with any other available tool. While the average number of mappings including a mismatch is high, 62 peptides were mapped to only a single locus, indicating true variant sequences. An example of a peptide mapped with a single amino acid substitution is highlighted with its sequence and the translated genomic sequence in Figure 11.

**Figure:**
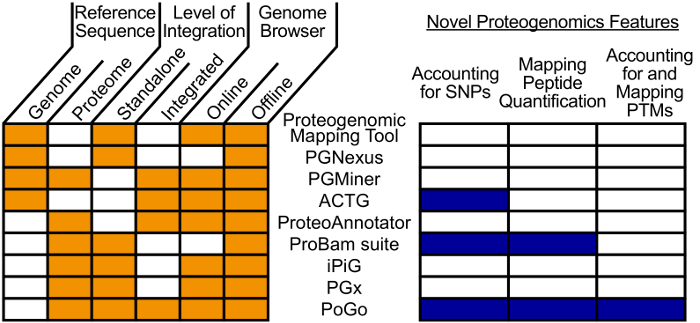

**Figure 1. Visual comparison of different peptide-to-genome mapping tools.** The comparison is shown with regards to various aspects. These aspects include a mapping reference, the level of integration into frameworks, and the support of online and offline browsers. Additionally, novel aspects of proteogenomics and their feature support is highlighted separately. PoGo only lacks the capability to directly map to a genome sequence compared to other tools. However, it supports all novel features that most of the other tools do not support. Please click here to view a larger version of this figure.

**Figure:**
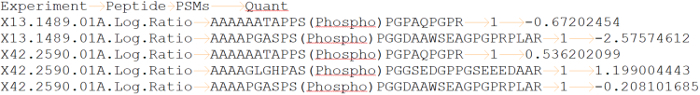

**Figure 2. Example input file for mapping peptides.** PoGo accepts input data in a tab-separated format with 4 columns. Column headers in the first line are 'Experiment', 'Peptide', 'PSMs', and 'Quant', indicating in the following lines the experiment or sample identifier, the peptide sequence, the number of peptide-spectrum matches, and a quantitative value for the peptide, respectively. File name extensions supported are *.txt, *.tsv, and *.pogo. Please click here to view a larger version of this figure.

**Figure:**
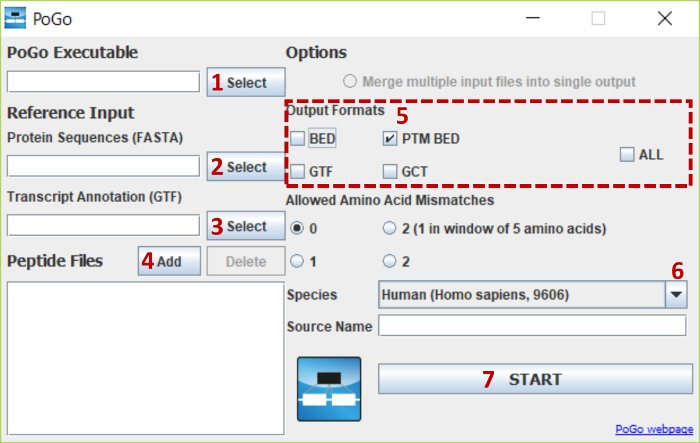

**Figure 3. PoGoGUI interface with highlighted steps for file selections and parameter options.** The figure shows the steps for selecting and uploading all required files and the selection of options for mapping peptides with post-translational modifications onto the human reference genome. Please click here to view a larger version of this figure.

**Figure:**
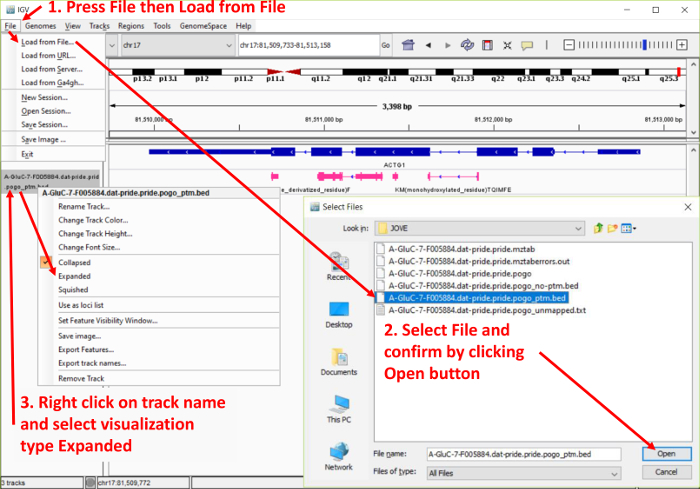

**Figure 4. Screenshot of the Integrative Genomics Viewer (IGV) data upload procedure.** The figure highlights the steps for uploading PoGo output files in the IGV browser. Furthermore, it shows the option of expanding the track of mapped peptides to highlight the mapping and sequence. Please click here to view a larger version of this figure.

**Figure:**
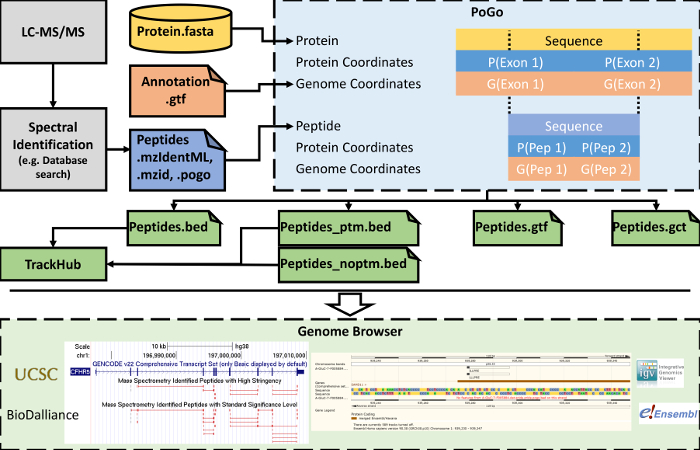

**Figure 5. Simplified workflow of steps from LC-MS/MS to visualization in genome browsers.** PoGo mapping follows the identification of peptides from tandem mass spectra. To achieve the mapping to the genome, PoGo utilizes reference annotation provided as genome annotation (GTF) and transcript translation sequences (FASTA). Different output formats are generated that can be loaded separately in genome browsers. Additionally, files in BED format can be combined into track hubs supporting visualization of large-scale datasets. Please click here to view a larger version of this figure.

**Figure:**
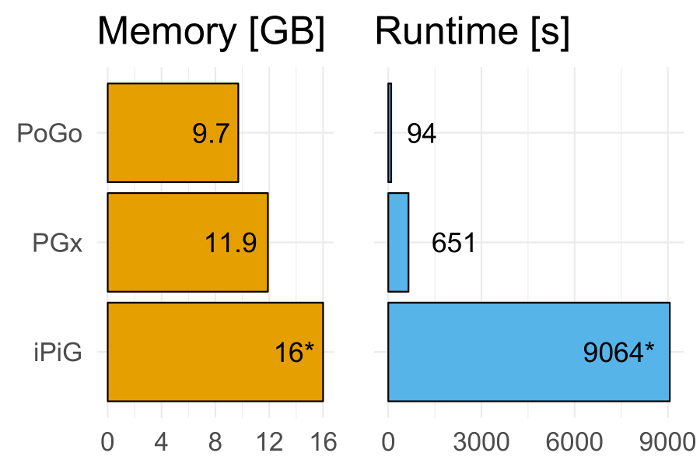

**Figure 6. Benchmarking PoGo against PGx and iPiG. PoGo outperforms the other tools on benchmarking.** Mapping 233,055 unique peptides across 59 adult and fetal tissues resulting in over 3 million sequences, PoGo was 6.9x and 96.4x faster than PGx and iPiG, respectively. Furthermore, PoGo required 20% and 60% less memory compared to PGx and iPiG, respectively. While PoGo and PGx finished successfully, iPiG resulted in a memory error at 16 GB. Please click here to view a larger version of this figure.

**Figure:**
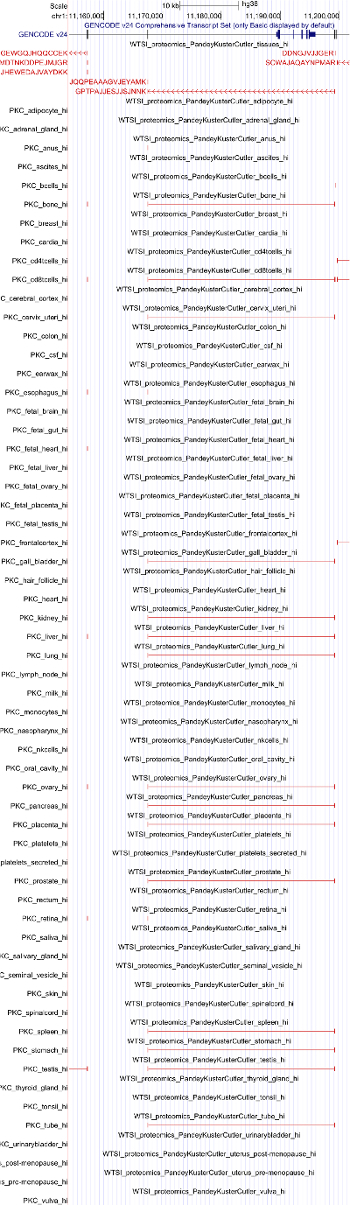

**Figure 7. UCSC Genome browser example view of mapped peptides.** The figure shows peptides mapped to the gene mTOR. While the combined track shows the peptides spanning across splice junctions and mapping only to one exon with the associated sequences, the tissue-specific tracks only highlight the mapping in a condensed format. Please click here to view a larger version of this figure.

**Figure:**
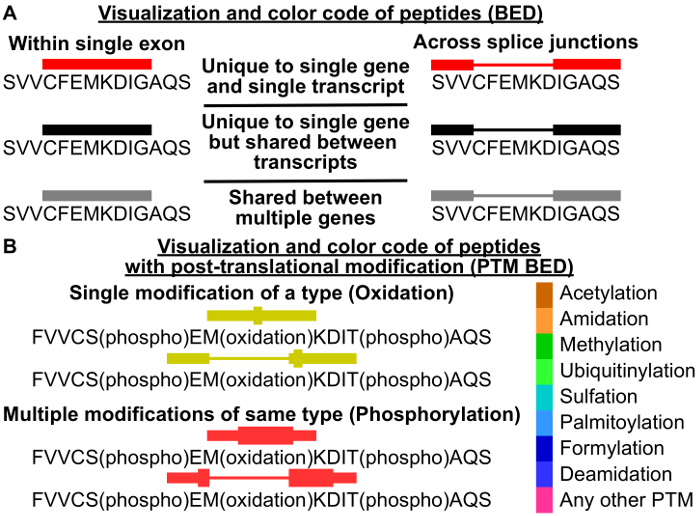

**Figure 8. Schematic of mapping visualization and color coding.** (**A**) In the standard BED output file, peptides mapping to an exon are shown as single blocks (left), while peptides mapping across multiple exons highlight the exon covering parts as blocks (right). Introns are shown as thin concatenating lines. PoGo color-codes the uniqueness of mapping or peptides to genes, and transcripts using a 3-tier system. (**B**) In addition to the block structure of the BED format, PTM BED output highlights the position of post-translational modifications as thick blocks. The presence of a single PTM of a type highlights the modified amino acid residue with a thick block, while multiple sites of the same PTM are combined into long blocks spanning from the first to the last modification site. Peptide mappings are further divided by PTM type and color codec based on the modification. Please click here to view a larger version of this figure.

**Figure:**
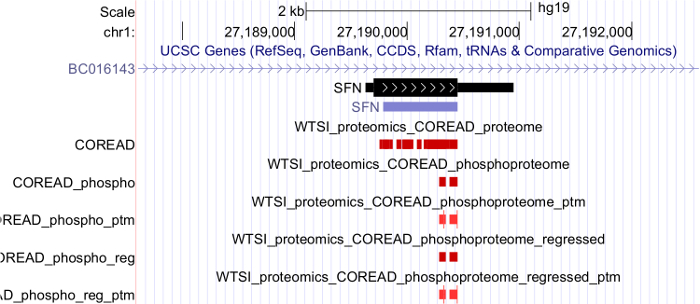

**Figure 9. Track hub view in the UCSC genome browser of colorectal cancer proteome and phosphoproteome data.** The track hub comprises whole proteome data as well as phosphoproteome. While the red color in the proteome and phosphoproteome tracks indicate the uniqueness of the mapping to the single transcript of SFN, tracks ending in _ptm show the phosphorylation sites within peptides. Here, the red color indicates the type of modification as phosphorylation. Only two peptides have been identified with each showing a single phosphorylation (thick blocks). Please click here to view a larger version of this figure.

**Figure:**
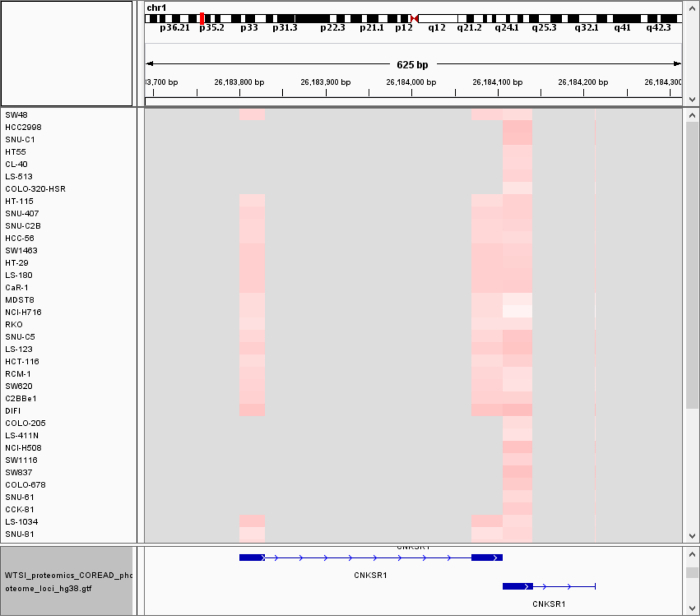

**Figure 10. View of colorectal cancer phosphopeptides and associated quantitation in IGV.** The figure shows a subset of the 50 cancer cell lines. It furthermore shows four columns of blocks in differing shades of light red. The color indicates the relative abundance from low (white) to high (red). While the four columns might initially lead to believe that there are 4 peptides, it becomes clear with the associated sequence-based GTF output file that these are in fact two peptides, each spanning a splice junction. Please click here to view a larger version of this figure.

**Figure:**
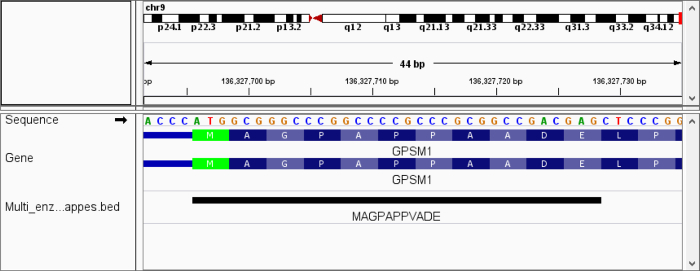

**Figure 11. View of peptide with amino acid variant in IGV.** The figure shows a peptide with a single amino acid variant mapped to the reference genome at the translation start of the gene* GPSM1*. The variant is positioned at amino acid residue 8 and results in the substitution of alanine to valine (A→V). The translation sequences of the annotated transcripts (blue) highlight the variant in comparison to the peptide sequence. Please click here to view a larger version of this figure.

## Discussion

This protocol describes how the software tool PoGo and its graphical user interface PoGoGUI enable a fast mapping of peptides onto genome coordinates. The tool offers unique features such as quantitative, post-translational modification and variant-enabled mapping to genomes using reference annotation. This article demonstrates the method on a large-scale proteogenomic study and highlights its speed and memory efficiency compared to other available tools^18^. In combination with the tool TrackHubGenerator, which creates online accessible hubs of genomic and genome linked data, PoGo, with its graphical user interface, enables large-scale proteogenomics studies to quickly visualize their data in genomic context. Furthermore, we demonstrate the unique features of PoGo with datasets searched against variant databases and quantitative phosphoproteomics^22^,^29^.

Single files, such as the GCT file, provide valuable visualization and links between peptide features and genomic loci. However, it is important to note that an interpretation based on these alone may be difficult or misleading due to their limitation to single aspects of proteogenomics such as uniqueness, post-translational modifications, and quantitative values. Therefore, it is important to carefully choose which output files, options, and combinations are appropriate for the proteogenomic question at hand and modify the combinations. For example, information about the uniqueness of the mapping to a specific genomic locus might be of great value for the annotation of a genomic feature^7^, while the quantification across different samples might be more appropriate for studies relating genomic features to changes in protein abundance^29^. The Output should be generated by PoGo for each setting. In case no output is generated, or empty files are shown in the output folder, it is recommended to check the input files for the desired content and the required file format. In cases where the file format or content does not follow the expectations of PoGo (*e.g.*, the FASTA file supposedly containing the transcript translation sequences contains the nucleotide sequences of the transcripts), error messages will ask the user to check the input files.

Restrictions of the protocol and the tool are mostly based on the reuse of file formats commonly used in genomics. Repurposing file formats used in genomics for proteogenomic applications is accompanied by specific limitations. These are due to the differing sets of requirements for genome centered visualization of genomic and proteogenomic data, such as the need to visualize post-translational modifications from proteomics data. This is restricted in the genomics file formats by single feature usage. Many approaches and tools have been developed for proteomics to confidently localize post-translational modifications within peptide sequences^31^,^32^,^33^,^34^. However, the visualization of multiple modifications in a unique and discernable manner on the genome is hindered by the structure of genomic file formats. Therefore, the single block visualization of multiple PTMs of the same type does not constitute any ambiguity of the modification sites but is the consequence of the differing requirement from the genomics community to only visualize single features at a time. Nonetheless, PoGo has the advantage of mapping post-translational modifications onto genomic coordinates to enable studies focused on the effect of genomic features such as single nucleotide variants on post-translational modifications. Using PoGo, variant mapping increases the number of total mappings. However, the unique color coding of mapped peptides highlights reliable mappings from unreliable ones. The mapping of variant peptides identified from known single nucleotide variants can be accompanied by visualizing the mapped peptides alongside the variants in VCF format. This way the color code indicating an unreliable mapping of a variant peptide is overruled by the presence of the known nucleotide variant.

A critical step for using PoGo is the use of the correct files and formats. The use of translated transcript sequences as protein sequences to accompany the annotation in GTF format is the main criteria. Another critical element when considering using PoGo to map peptides with amino acid mismatches is memory. While highly memory-efficient for a standard application, the significantly and exponentially increasing number of possible mappings with one or two mismatches leads to a similarly exponential increase in memory usage^18^. We propose a staged mapping as described in this protocol to first map the peptides without mismatches and remove them from the set. The subsequent previously unmapped peptides then can be mapped using one mismatch and the procedure can be repeated with two mismatches for the peptides remaining unmapped.

Since the throughput of mass spectrometry has significantly increased and studies interfacing genomic and proteomic data are becoming more frequent in recent years, tools to readily enable interfacing these types of data in the same coordinate system are increasingly indispensable. The tool presented here will aid the need to combine genomic and proteomic data to enhance a better understanding of integrative studies across small and large datasets by mapping peptides onto a reference annotation. Encouragingly, PoGo has been applied to map peptides to gene candidates provided in the same format as the reference annotation to support annotation efforts of novel genes expressed in human testis^35^. The approach presented here is independent of databases used for peptide identification. The protocol might aid in the identification and visualization of novel translation products by using adapted input files from translation sequences and associated GTF files from RNA-seq experiments.

Several approaches and tools with a wide range of special application scenarios to map peptides to genomic coordinates, ranging from mapping peptides directly to the genome sequence to RNA-sequencing guided mapping, have been introduced^10^,^11^,^12^,^13^,^14^,^15^,^16^,^17^. However, these can result in a failure to correctly map peptides when post-translational modifications are present and errors in the underlying mapping of RNA-sequencing reads may be propagated to the peptide level. PoGo has been developed to specifically overcome those obstacles and to cope with the rapid increase of quantitative high-resolution proteomic datasets to integrate with orthogonal genomics platforms. The tool described here can be integrated into high-throughput workflows. Through the graphical interface PoGoGUI, the tool is simple to use and requires no specialist bioinformatics training.

## Disclosures

The authors have nothing to disclose.
